# Ability of the ALBI grade to predict posthepatectomy liver failure and long-term survival after liver resection for different BCLC stages of HCC

**DOI:** 10.1186/s12957-018-1500-9

**Published:** 2018-10-16

**Authors:** Ze-Qun Zhang, Li Xiong, Jiang-Jiao Zhou, Xiong-Ying Miao, Qing-Long Li, Yu Wen, Heng Zou

**Affiliations:** Department of General Surgery, The Second Xiangya Hospital, Central South University, Changsha, 410011 Hunan China

**Keywords:** Child-Pugh grade, Post-hepatectomy liver failure, Albumin-bilirubin score, Hepatocellular carcinoma, BCLC classification, Overall survival

## Abstract

**Background:**

Underlying liver function is a major concern when applying surgical resection for hepatocellular carcinoma (HCC). We aimed to explore the capability of the albumin-bilirubin (ALBI) grade to predict post-hepatectomy liver failure (PHLF) and long-term survival after hepatectomy for HCC patients with different Barcelona Clinic Liver Cancer (BCLC) stages.

**Methods:**

Between January 2010 and December 2014, 338 HCC patients who were treated with liver resection were enrolled. The predictive accuracy of ALBI grade system for PHLF and long-term survival across different BCLC stages was examined.

**Results:**

A total of 26 (7.7%) patients developed PHLF. Patients were divided into BCLC 0/A and BCLC B/C categories. ALBI score was found to be a strong independent predictor of PHLF across different BCLC stages by multivariate analysis. In terms of overall survival (OS), it exhibited high discriminative power in the total cohort and in BCLC 0/A subgroup. However, differences in OS between ALBI grade 1 and 2 patients in BCLC B/C subgroup were not significant (*P* = 0.222).

**Conclusion:**

The ALBI grade showed good predictive ability for PHLF in HCC patients across different BCLC stages. However, the ALBI grade was only a significant predictor of OS in BCLC stage 0/A patients and failed to predict OS in BCLC stage B/C patients.

**Electronic supplementary material:**

The online version of this article (10.1186/s12957-018-1500-9) contains supplementary material, which is available to authorized users.

## Background

As one of the most common and aggressive malignancies, hepatocellular carcinoma (HCC) ranks the second most fatal cancer globally [1]. Although surgical resection is the mainstay therapy for extremely early and early stage HCC, it is not recommended for intermediate and advanced stage HCC as determined by Barcelona Clinic Liver Cancer (BCLC) classification because of the increased risks and limited advantages of the procedure [2]. Recently, due to advances in techniques and perioperative management, surgical resection for HCC has become more aggressive. HCC patients at BCLC B and C stages have been reported to have good prognosis after surgical resection [3–6].

Patients with BCLC B and C HCC often possess a high tumor burden, including an increased tumor size, multiple tumors, and vascular invasion. To achieve microscopically radical resection margins, patients tend to undergo extended liver resection, which increases the chances of post-hepatectomy liver failure (PHLF). This is especially obvious when patients have underlying long-term hepatic disorders, such as hepatic fibrosis and cirrhosis [7–9], which often result in impaired liver function. As a life-threatening complication with an intrinsic risk of mortality, PHLF is still a major concern for hepatic surgeons in clinical practice. To minimize PHLF and postoperative mortality, development of a simple, objective, and accurate assessment tool for liver function prior to surgery is of vital importance. Recently, the application of albumin-bilirubin (ALBI) grade to evaluate liver function in patients with HCC was proposed [10–12]. The ALBI grade not only offers similar prognostic information as the Child-Pugh (CP) class but also eliminates the necessity of assessing empirical variables such as hepatic encephalopathy and ascites. Several studies have shown that the ALBI grade had a superior predictive value for PHLF and overall survival (OS) in HCC patients following liver resection [13, 14].

The survival of HCC patients undergoing liver resection depends mainly on tumor burden and hepatic function [15]. The ALBI grade was proven to be a reliable hepatic function assessment tool and could accurately predict survival in patients with extremely early or early stage HCC who underwent liver resection [12, 16–19]. However, few studies have examined the role of the ALBI grade in predicting the survival of patients with intermediate and advanced stage HCC after hepatectomy.

Here, we evaluated the effectiveness of the ALBI grade in predicting PHLF and OS across different BCLC stages among HCC patients undergoing liver resection.

## Methods

### Patients

In this retrospective study, HCC patients who underwent liver resection were enrolled from January 2010 to December 2014 at the Second Xiangya Hospital. The hospital medical database was searched to retrieve baseline parameters. Inclusion criteria included the following: liver function with CP A or B; no other simultaneous malignancies; no therapy for HCC before surgery; and no insufficiency concerning the heart, lung, kidney, and brain before operation. Written informed consent for this retrospective research was waived. This investigation was approved by the ethics committee of the hospital.

### Diagnosis and definitions

HCC was diagnosed using histopathological examination of the surgical samples. The HCC stage accorded with the BCLC guidelines [20]. Based on the recommendations of the International Study Group of Liver Surgery (ISGLS), PHLF was defined as a total serum bilirubin value > 50 μmol l^− 1^ on day 5 after surgery or hereafter and a prothrombin time index < 50% (an international normalized ratio (INR) > 1.7) in the meanwhile [21, 22]. Adjustment in clinical administration was not required for grade A PHLF; non-invasive interventions such as fresh-frozen plasma, albumin management, diuretics, and ventilation were applied for grade B PHLF; and invasive interventions such as circulatory and extracorporeal liver support, hemodialysis, intubation, and mechanical ventilation were used barely for grade C PHLF [21]. A relative low platelet count (< 100 × 10^9^/l) with splenomegaly and diagnosis of gastric/esophageal varices were used as indicators of clinically significant portal hypertension (CSPH) [23, 24]. Hepatectomy was defined as minor if fewer than three Couinaud segments were resected and major if three liver segments or more were resected. Deaths recorded within 60 days after surgery were considered to represent mortality.

Determination of the CP class was performed according to methods published previously [25]. The model of end-stage liver disease (MELD) score was computed applying the formula: 9.57 × ln(creatinine [mg/dl]) + 11.2 × ln(INR) + 3.78 × ln(bilirubin [mg/dl]) + 6.43 [26]. The calculation of the ALBI score used this equation: ALBI score = − 0.085 × (albumin [g l^− 1^]) + 0.66 × log_10_(total bilirubin [μmol l^− 1^]), and was further categorized into three different grades: grade 3 (> − 1.39), grade 2 (> − 2.60 to ≤ − 1.39), and grade 1 (≤ − 2.60) [10].

### Surgical technique

Before surgery, contrast-enhanced computed tomography (CT) and abdominal ultrasound were routinely conducted to assess tumor condition. Preoperative indocyanine green tests were routinely performed for patients with hepatitis B or C infection. For laparotomy, right subcostal margin incision was chosen. Laparoscopic approach was implemented widely for tumor diameter < 5 cm located in segments 2–6. During operation, the fluid infusion was minimal to maintain a relatively low central venous pressure to reduce bleeding. To precisely determine the relationship between tumors and other tissues as well as assess the orientation of tumors, intraoperative ultrasound was conducted if needed. If resectability had been determined, anatomical resection was performed aiming to excise the tumor’s portal territory when future remnant liver functional reserve was sufficient.

### Follow-up evaluation

The trace of each patient was done at 1 month following discharge from hospital and at intervals of 3 months during the first year, and at 6-month intervals in following years. Follow-up included liver function tests, determination of α-fetoprotein (AFP) concentration, and abdominal-enhanced CT. The primary endpoints for this study were PHLF and death. OS was from date of resection to last living visit or loss to follow-up. The last visit was done on December 31, 2017.

### Statistical analysis

Continuous data were analyzed by *t* test or Mann-Whitney *U* test. *χ*^*2*^ test was used to compare categorical data. Identification of independent predictors of PHLF was achieved by multivariate logistic regression analysis. Receiver operating characteristic (ROC) curve analysis was carried out to determine the cut-off points for the occurrence of PHLF. The area under the ROC curve (AUC) was applied to assess discriminative power. Comparisons between ROC curves were conducted with Delong test. OS was assessed visually through Kaplan-Meier plots, and differences between curves were analyzed by log-rank test. Independent risk factors for OS were identified using multivariate Cox proportional hazard regression models. Statistical significance was considered for a two-tailed value of *P* < 0.05. SPSS 17.0 (Inc., Chicago, IL, USA) was applied for data analysis.

## Results

### Patient information

A sample of 338 HCC patients was included. Most patients had a CP class of A (308/338, 91.1%), and the remaining 30 were class B (30/308, 8.9%). For ALBI grade 1, there were 39.6% (134/338) patients while grade 2 had 58.6% patients (198/338) and grade 3 had 1.8% (6/338) patients. Table 1 shows the features of the patients.

**Table 1:** Baseline characteristics of 338 HCC patients

Variables	Total cohort (*n* = 338)	BCLC 0/A HCC (*n* = 205)	BCLC B/C HCC (*n* = 133)
Age, years^†^	52 (44–66)	52 (44–60)	52 (43–60)
Male gender^‡^	299 (88.5)	180 (87.5)	119 (89.5)
Positive HBsAg^‡^	278 (82.2)	170 (82.9)	108 (81.2)
Total bilirubin, μmol/l^†^	14.0 (13.4–19.4)	13.9 (10.0–19.6)	14.4 (10.6–18.7)
Albumin, g/l^†^	37.9 (35.1–40.8)	38.0 (35.4–41.0)	37.6 (34.0–40.7)
ALT, U/l^†^	36.1 (25.8–52.0)	34.3 (24.7–53.0)	37.9 (26.4–51.0)
Prothrombin time, s^†^	13.2 (12.0–14.1)	13.2 (12.0–14.1)	13.2 (12.1–14.1)
INR^†^	1.04 (0.95–1.14)	1.04 (0.94–1.14)	1.05 (0.95–1.14)
Platelet count, × 10^9^/l^†^	155 (110–205)	155 (110–211)	154 (113.5–201.5)
Maximum tumor size, cm^†^	6.0 (4.2–10.0)	6.0 (3.5–9.2)	8 (5.0–11.0)
Serum AFP, ng/ml^‡^			
≥ 400	141 (41.7)	73 (35.6)	68 (51.1)
< 400	197 (58.3)	132 (64.4)	65 (48.9)
CSPH^‡^	56 (16.6)	34 (16.6)	22 (16.5)
ALBI score^†^	− 2.460 (− 2.704– − 2.192)	− 2.518 (− 2.719–− 2.236)	− 2.399 (− 2.702–− 2.136)
ALBI grade^‡^			
1	134 (39.6)	89 (43.4)	45 (33.8)
2	198 (58.6)	114 (55.6)	84 (63.2)
3	6 (1.8)	2 (1.0)	4 (3.0)
MELD score (range)	7 (6–18)	7 (6–18)	7 (6–17)
MELD score^‡^			
≥ 9	88 (26.0)	47 (22.9)	41 (30.8)
< 9	250 (74.0)	158 (77.1)	92 (69.2)
Child-Pugh grade^‡^			
A	308 (91.1)	190 (92.7)	118 (88.7)
B	30 (8.9)	15 (7.3)	15 (11.3)
C	0 (0)	0 (0)	0 (0)
BCLC stage^‡^			
0	12 (3.6)	–	–
A	193 (57.1)	–	–
B	82 (24.3)	–	–
C	51 (15.1)	–	–

*HCC* hepatocellular carcinoma, *HBsAg* hepatitis B surface antigen, *ALT* alanine aminotransferase, *INR* international normalized ratio, *AFP* α-fetoprotein, *CSPH* clinically significant portal pressure, *ALBI* albumin-bilirubin, *BCLC* Barcelona Clinic Liver Cancer

†Values are median (interquartile range)

‡Values are number (%)

### Postoperative morbidity, PHLF, and mortality

Of the 338 patients, 142 (42.0%) developed complications after surgery. Pneumonia which had the highest frequency occurred in 39 patients (11.5%), followed by plural effusion and ascites in 37 (10.9%). PHLF occurred in 26 patients (7.7%), among them, 8 patients (2.4%) had grade A PHLF, 13 patients (3.8%) had grade B, and 5 patients (1.5%) had grade C. During 60 days after operation, 15 patients died, resulting in a postoperative mortality rate of 4.4%.

### Correlation between PHLF and CP class or ALBI grade

**Fig. 1:**
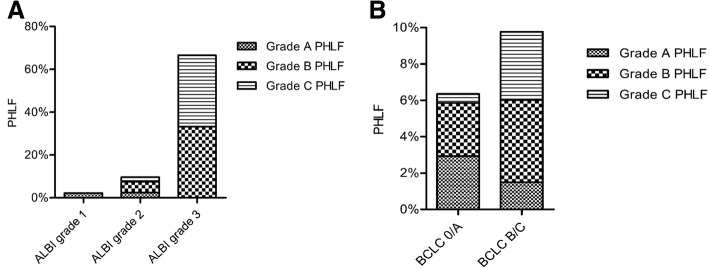
Correlation between incidence and severity of PHLF and ALBI grade (**a**), and BCLC classification subgroups (**b**). PHLF, posthepatectomy liver failure; BCLC, Barcelona Clinic Liver Cancer

PHLF occurred in 16 of the 308 (5.2%) CP class A patients and in 10 of the 30 (33.3%) CP class B patients (*P* < 0.001). Among the total cohort, 3 of 134 (2.2%) ALBI grade 1 patients had PHLF, while 19 of 198 (9.6%) ALBI grade 2 patients fell into PHLF (*P* = 0.008). Four of 6 (66.7%) ALBI grade 3 patients developed PHLF. Higher ALBI grade led to higher risk of grade C PHLF (Fig. 1a). The incidence of grade C PHLF was higher in patients with ALBI grade 3 compared to those with ALBI grade 2 (*P* = 0.003) or ALBI grade 1 (*P* = 0.001). Moreover, patients with BCLC B/C HCC were more likely to suffer from grade C PHLF than those with BCLC 0/A HCC (*P* = 0.027, Fig. 1b).

### Multivariate analyses of PHLF across the BCLC stages of HCC

The entire cohort was categorized into two subgroups in the multivariate logistic regression analysis. For the total cohort, ALBI score, CP score, and major hepatectomy were identified significant. In the BCLC 0/A subgroup, ALBI score, CP score, and major hepatectomy were identified significant. In the BCLC B/C subgroup, platelet count, ALBI score, and CP score were identified significant (Table 2).

**Table 2:** Multivariate logistic regression analyses for posthepatectomy liver failure across BCLC stages

Variable	Multivariate logistic regression					
	Total cohort		BCLC 0/A		BCLC B/C	
	OR (95% CI)	*P*	OR (95% CI)	*P*	OR (95% CI)	*P*
Prothrombin time > 14 s	1.19 (0.39–3.63)	0.765	1.39 (0.35–5.47)	0.635	0.48 (0.04–6.60)	0.584
Platelet count < 100 × 10^9^/l	2.82 (0.97–8.19)	0.056	0.45 (0.07–2.94)	0.405	132.70 (6.04–916.91)	0.002
Tumor size > 5 cm	0.65 (0.21–2.01)	0.456	0.50 (0.11–2.23)	0.361	0.49 (0.04–6.52)	0.587
ALBI score > −2.44	3.43 (1.11–10.57)	0.032	2.33 (1.09–9.19)	0.046	30.48 (1.36–682.73)	0.031
Child-Pugh score > 6	6.47 (2.10–19.94)	0.001	5.50 (1.23–24.50)	0.025	43.21 (2.43–767.29)	0.010
Blood loss > 400 ml	2.13 (0.83–5.44)	0.115	1.75 (0.55–5.57)	0.346	5.86 (0.53–64.72)	0.149
Major hepatectomy	5.51 (1.86–16.29)	0.002	7.23 (1.73–30.57)	0.007	12.02 (0.97–149.04)	0.053
MELD score > 8	0.67 (0.21–2.11)	0.496	0.90 (0.21–3.78)	0.885	0.35 (0.03–3.62)	0.378

*OR* odds ratio, *CI* confidence interval, *BCLC* Barcelona Clinic Liver Cancer, *ALBI* albumin-bilirubin, *MELD* model for end-stage liver disease

### Discriminative power of ALBI score to predict PHLF across the BCLC stages of HCC

**Fig. 2:**
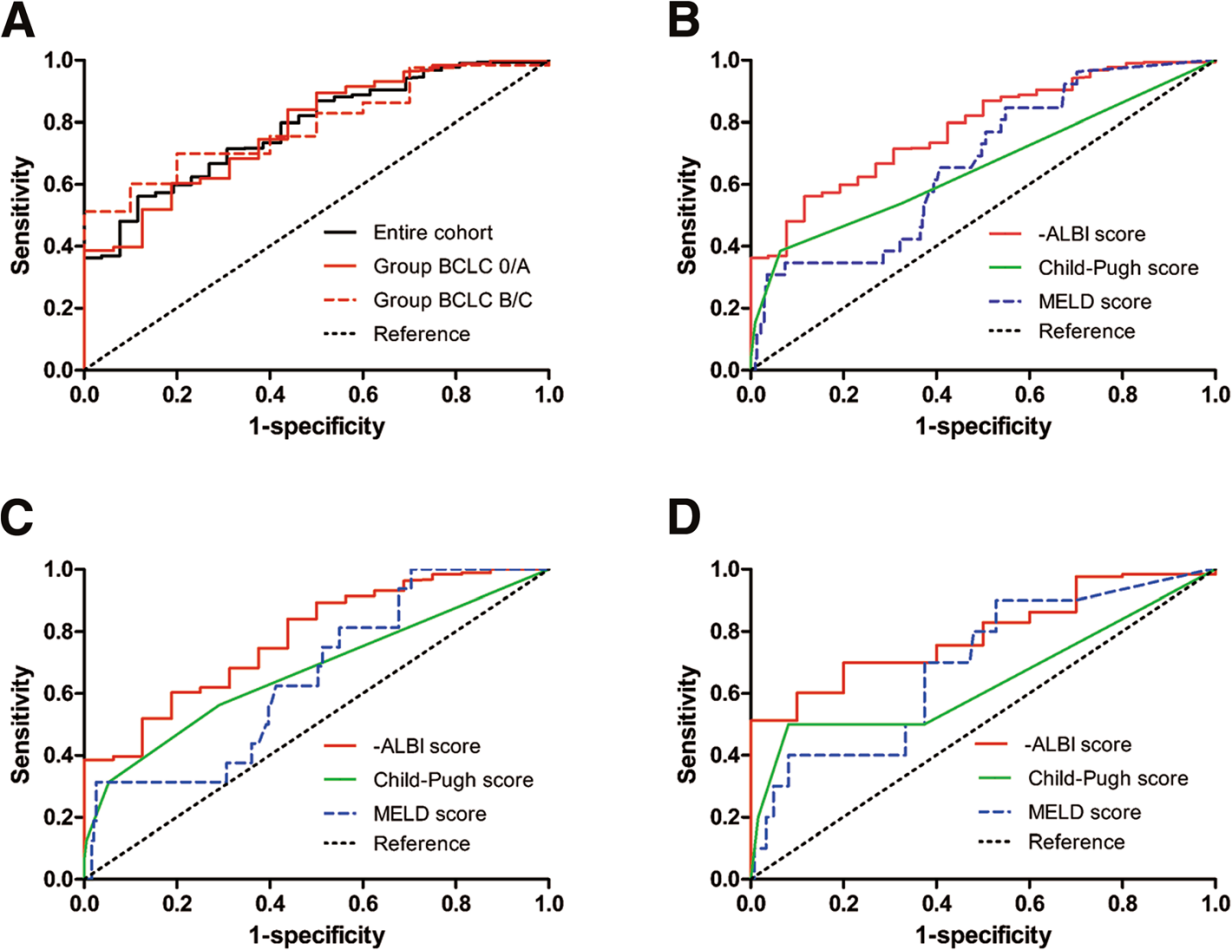
Receiver operating characteristic (ROC) curve analyses of -ALBI scores for predicting PHLF in the entire cohort and the BCLC stage subgroups (**a**). ROC curves for Child-Pugh score, MELD score, and -ALBI score for predicting PHLF in the entire cohort (**b**), BCLC 0/A subgroup (**c**), and BCLC B/C subgroup (**d**). ALBI, albumin-bilirubin; BCLC, Barcelona Clinic Liver Cancer; PHLF, posthepatectomy liver failure. MELD, model for end-stage liver disease

Figure 2a shows the predictive power of -ALBI scores for PHLF as determined by the ROC curve analyses which were the total cohort (AUC, 0.782; 95% CI, 0.701–0.862, *P* < 0.001), BCLC 0/A subgroup (AUC, 0.780; 95% CI, 0.670–0.889; *P* < 0.001), and BCLC B/C subgroup (AUC, 0.790; 95% CI, 0.680–0.900; *P* = 0.002). For the total cohort, the -ALBI score had a greater AUC than CP score (AUC, 0.656; 95% CI, 0.527–0.784; *P* = 0.008) (*P* = 0.005, Delong test) and MELD score (AUC, 0.669; 95% CI, 0.566–0.771; *P* = 0.004) (*P* = 0.013, Delong test) (Fig. 2b). The -ALBI score had an optimal cut-off value of 2.44 (that was to say, the cut-off point of ALBI score lay in − 2.44, the same below), presenting a specificity and a sensitivity of 56.1% and 88.5% respectively.

We divided the entire cohort into two subgroups, BCLC 0/A and BCLC B/C, to minimize the potential confounding bias caused by tumor characteristics. The cut-off value of the former group was 2.41, with a specificity and a sensitivity of 60.3% and 81.3% respectively, while the cut-off value of the latter group was 2.36, with a sensitivity of 90.0% and a specificity of 60.2%. In the BCLC 0/A group, the AUC of -ALBI score was greater than MELD and CP scores (Fig. 2c). Same result could be obtained in BCLC B/C subgroup (Fig. 2d).

### Discriminative power of the ALBI grade for OS at different BCLC stages of HCC

**Fig. 3:**
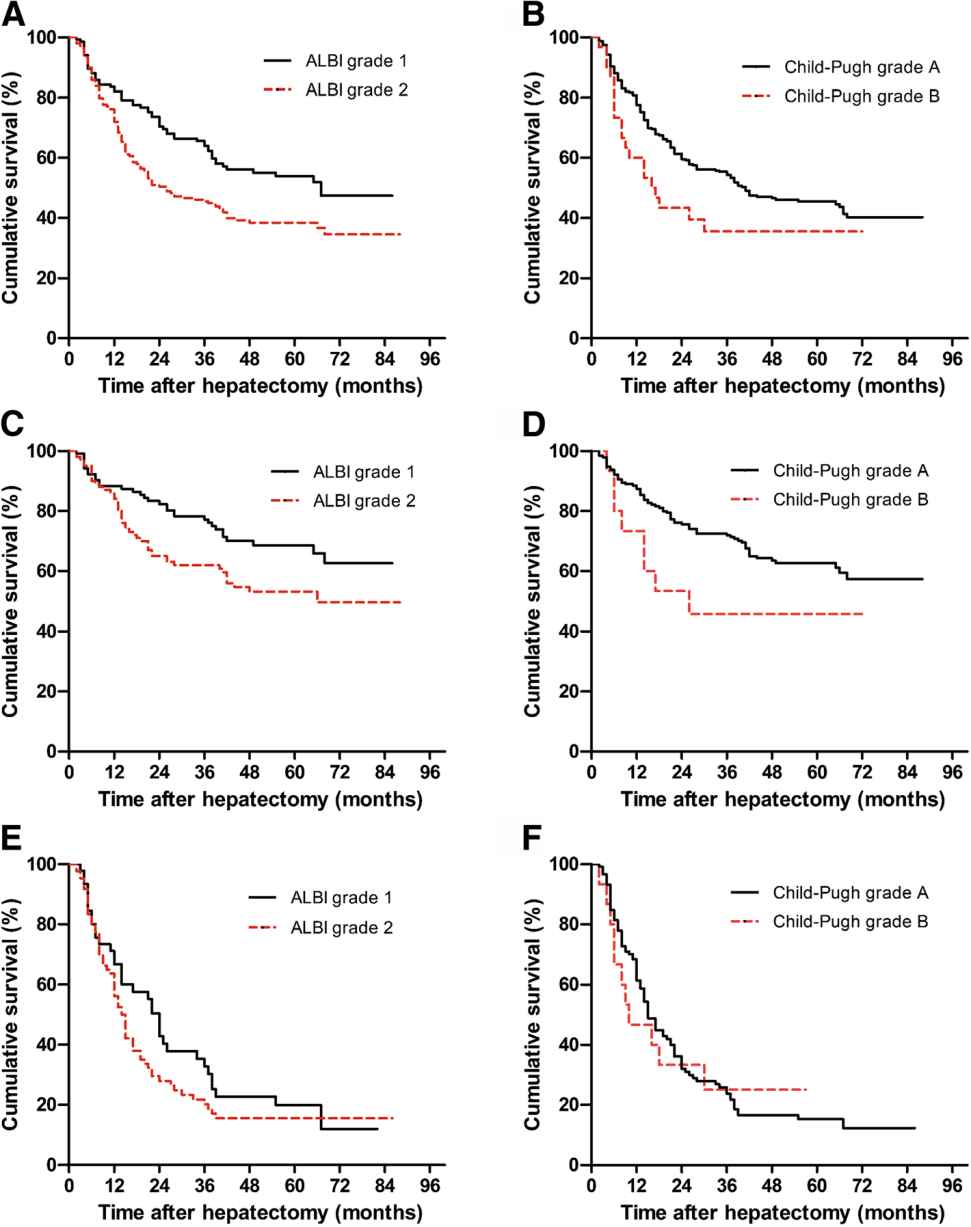
Kaplan-Meier curves demonstrating overall survival according to the **a** ALBI grade in the total cohort, **b** Child-Pugh class in the total cohort, **c** ALBI grade in the BCLC 0/A subgroup, **d** Child-Pugh class in the BCLC 0/A subgroup, **e** ALBI grade in the BCLC B/C subgroup, and **f** Child-Pugh class in the BCLC B/C subgroup. ALBI, albumin-bilirubin; BCLC, Barcelona Clinic Liver Cancer

A total of 183 HCC deaths (54.1%) occurred throughout the follow-up (with a median of 31.5 months). The OS rate in the total cohort at 1, 2, and 3 years were 78.1%, 55.3%, and 49.4%, in that order. The OS rate in patients with ALBI grade 1 at 1, 2, and 3 years were 83.6%, 68.7%, and 61.2%, respectively, which were higher compared to patients with ALBI grade 2 (74.7%, 47.0%, and 41.9%, respectively) (*P* = 0.003) (Fig. 3a). We did not analyze the OS of the ALBI grade 3 cohort because there were few patients (6 patients) in this group. Four of these patients died within 2 years after the operation. In the BCLC 0/A subgroup, the OS rate of ALBI grade 2 patients was lower compared to that of ALBI grade 1 patients (*P* = 0.008) (Fig. 3c). However, in BCLC B/C subgroup, OS did not differ between ALBI grade 2 patients and ALBI grade 1 patients (*P* = 0.222) (Fig. 3e). The OS exhibited little difference between patients with CP class A and those with CP class B in the total cohort (*P* = 0.052) (Fig. 3b), BCLC 0/A subgroup (*P* = 0.052) (Fig. 3d) and BCLC B/C subgroup (*P* = 0.911) (Fig. 3f). Moreover, comparison of OS between MELD scores < 9 and ≥ 9 also showed no statistical difference (*P =* 0.784) (Additional file 1: Figure S1).

### Multivariate cox regression analyses of OS across BCLC stages

Multivariate analyses revealed that the ALBI score, platelet count, tumor size, microvascular invasion (MVI), and differentiation grade were significant risk factors for OS in the total cohort. We further performed multivariate analysis in the two subgroups (Table 3). In the BCLC 0/A subgroup, we observed similar results as in the total cohort (Table 3). Interestingly, the ALBI score was not a significant predictor, only MVI and differentiation grade remained independent predictors of OS in the BCLC B/C subgroup (Table 3).

**Table 3:** Multivariate analyses of factors affecting overall survival across BCLC stage

Variable	Multivariable Cox regression					
	Total cohort		BCLC 0/A		BCLC B/C	
	HR (95% CI)	*P*	HR (95% CI)	*P*	HR (95% CI)	*P*
Age, years	1.03 (0.72–1.48)	0.868	1.08 (0.61–1.93)	0.791	0.79 (0.49–1.29)	0.343
Male gender	0.86 (0.53–1.40)	0.541	1.10 (0.51–2.37)	0.803	0.85 (0.44–1.65)	0.626
Platelet count, × 10^9^/l	1.62 (1.10–2.38)	0.014	1.90 (1.02–3.53)	0.043	1.26 (0.75–2.11)	0.383
Prothrombin time, sec	1.00 (0.89–1.12)	0.938	0.92 (0.76–1.11)	0.362	1.07 (0.92–1.25)	0.379
Serum AFP, ng/ml	1.09 (0.80–1.49)	0.594	1.22 (0.72–2.05)	0.457	0.92 (0.61–1.39)	0.677
Tumor size, cm	1.71 (1.21–2.44)	0.003	1.95 (1.15–3.32)	0.014	1.58 (0.95–2.64)	0.079
ALBI score	1.62 (1.18–2.22)	0.003	2.29 (1.39–3.78)	0.001	1.42 (0.91–2.20)	0.122
Major hepatectomy	1.33 (0.93–1.88)	0.115	1.02 (0.53–1.95)	0.965	1.33 (0.86–2.08)	0.201
MVI	2.10 (1.49–2.97)	< 0.001	1.91 (1.13–3.01)	0.028	1.53 (0.89–2.83)	0.046
Differentiation grade	1.69 (1.24–2.30)	0.001	1.77 (1.08–2.89)	0.024	2.03 (1.28–3.23)	0.003
MELD score	0.98 (0.90–1.08)	0.727	0.98 (0.84–1.15)	0.803	1.00 (0.88–1.14)	0.989

*HR* hazard ratio, *CI* confidence interval, *BCLC* Barcelona Clinic Liver Cancer, *AFP* α-fetoprotein, *ALBI* albumin-bilirubin, *MVI* microvascular invasion, *MELD* model for end-stage liver disease

## Discussion

Surgical resection is extensively performed for HCC patients with a favorable liver functional reserve. However, PHLF remains a serious complication of hepatic resection and a main cause of postoperative mortality [7, 27]. To improve the survival of patients with PHLF, early diagnosis and therapy are imperative. Many previous reports have provided evidence that the ALBI grade is an effective predictor of PHLF after liver resection in patients with HCC [13, 14]. However, few studies have focused on the role of the ALBI score in predicting PHLF for patients with intermediate and advanced HCC. We found that the ALBI score could predict PHLF not only in the total cohort but also in the BCLC 0/A and BCLC B/C subgroups. BCLC B/C patients have a relatively higher tumor burden and often possess a larger tumor size, satellite nodes, or portal/hepatic vein tumor thrombosis. To achieve a radical cure, patients are inclined to undergo extensive or major hepatectomy in clinical practice, which increases the probability of occurrence of PHLF, especially for patients suffering from chronic hepatic disorders. For these reasons, accurate assessment of hepatic function prior to surgery is of great clinical significance for these patients.

To determine whether ALBI score can predict PHLF accurately, we used a stage-stratified approach. Patients with BCLC B/C HCC owned a greater possibility of occurring severer PHLF than those with BCLC 0/A HCC. Several facts may contribute to this result. On the one hand, generally, BCLC B/C HCC own a heavier tumor burden, it is easy to make extensive hepatectomy to achieve radical resection. On the other hand, BCLC B/C HCC have a longer time of tumor progression, so the liver function of these patients might be worse than that of BCLC 0/A patients. ROC curve analyses demonstrated that the AUC values of MELD and CP scores were lower compared to that of the -ALBI score in predicting PHLF in the total cohort and the BCLC stage subgroups, implying that the ALBI score might have better prognostic value for PHLF among patients undergoing liver resection. This was consistent with previous reports [13, 14].

In addition, our results revealed that the -ALBI grade had an AUC of 0.790 for predicting PHLF in BCLC B/C patients, which indicates a relatively good prognostic value. The ROC curve showed that optimal cut-off value of -ALBI calculated by the ROC curve was 2.36 in BCLC B/C HCC subgroup. This suggested that for patients with an ALBI score > − 2.36 in BCLC B/C stage, increased attention should be given by hepatic surgeons to avoid PHLF. Additionally, the ALBI score showed a favorable prognostic value for PHLF in the total cohort and in the BCLC 0/A subgroup, which is consistent with the earlier studies [16, 17]. This study indicated that the ALBI score might be helpful in choosing eligible candidates who may benefit from surgery, especially among BCLC B/C patients with a small-sized liver after resection. Moreover, we found MELD score showed no significance in the multivariate analysis for PHLF in this research. On the one hand, this might be on account of the small sample size from a single center. On the other hand, MELD score maybe suffers some limitations itself. For example, several other studies reported that MELD score showed no predictive power for perioperative outcomes in HCC patients without cirrhosis [28–30]. And interestingly, we found that the platelet count was an effective predictor of PHLF only in BCLC B/C patients, but not in BCLC 0/A patients. The platelet count is commonly thought to be correlated with the severity of portal hypertension [31]. Therefore, portal hypertension might play a more important role in predicting PHLF for BCLC B/C patients than in those with early stage HCC, but further study is required to verify this conclusion.

The survival of HCC patients was partly impacted by the underlying liver function. Consistent with the previous studies [16–18], we found that the ALBI score was a significant risk factor for OS in both the total cohort and the BCLC 0/A subgroup. Moreover, the OS rate was higher in ALBI grade 1 patients compared to that in ALBI grade 2 patients both in the total cohort and in the BCLC 0/A subgroup. However, OS showed little difference among CP classes in the total cohort and the two subgroups. These results suggested that the ALBI grade might have a greater discriminative ability than the CP class for predicting the OS of HCC patients after liver resection in a curative setting.

Furthermore, the prognostic value of ALBI scores was evaluated for BCLC B/C patients. According to the BCLC staging system in most Western countries, hepatectomy is not recommended for this unique group of patients [20]. However, recent studies have demonstrated that intermediate and advanced HCCs were not absolute contradictions for surgical resection, which could provide significant survival benefits for carefully selected patients [6]. Importantly, we found that OS showed little difference between ALBI grade 2 and ALBI grade 1 patients in this cohort (*P* = 0.222). Moreover, multivariate Cox regression model revealed that the ALBI score showed no predictive ability in the BCLC B/C subgroup (*P* = 0.122).

Prognoses of HCC patients are largely dependent on the tumor characteristics at the time when they undergo surgery. We found differentiation grade and MVI were significant predictors in the Cox regression model. Compared to patients with early stage HCC, patients with BCLC B/C HCC generally have heavier tumor burdens, as indicated by large tumor sizes, poor differentiation, and vascular invasion [32]. Vascular invasion is an independent risk factor of recurrence and OS, directly correlated with the size of the main nodule and histological differentiation [33, 34]. Differentiation grade indicates a greater likelihood of malignant behavior, resulting in a higher risk of recurrence and metastasis, significantly affect OS. Interestingly, AFP level showed no significance in predicting OS in our study, despite this marker has been reported to predict reaction to locoregional treatments and outcome of untreated advanced HCC [35, 36]. To summarize, tumor burden is likely to play a more significant role in determining survival time than the underlying liver function in this group of patients. Thus, the ALBI grade alone may not be sufficient to predict OS in BCLC B/C stage HCC patients who undergo liver resection. A possible solution may be integrating the ALBI grade and tumor characteristics to generate a more accurate model to predict OS for this population, which should be performed in the future.

Several limitations exist in the present research. First, most of the patients had an infection of hepatitis B virus as the cause of HCC in the current research. Our results may not be applicable to Western countries, where hepatitis C virus infection and alcoholic steatohepatitis are the predominant etiologies of HCC. Second, the reliability of the present research was weakened by its retrospective specialty, single-center data, and comparably small sample capacity. Third, limited by the relatively small sample size, we did not include some variables which might have an effect on PHLF in order to increase the stability of statistical models and the credibility of results. A prospective study design with a large sample and effective controls is needed to expand our findings.

## Conclusions

In summary, our study has verified that the ALBI grade is a significant prognostic factor for PHLF in HCC patients across different BCLC stages. However, for the first time, we found the ALBI grade was only a significant predictor of OS in BCLC stage 0/A patients and was not a good predictor in BCLC stage B/C patients.

## Additional file

Additional file 1: **Figure S1.** Kaplan-Meier curves demonstrating overall survival according to MELD score < 9 and MELD score ≥ 9 in the total cohort. BCLC, Barcelona Clinic Liver Cancer; MELD, model for end-stage disease. (TIF 801 kb)

## Abbreviations

AFP: α-fetoprotein
ALBI: Albumin-bilirubin
BCLC: Barcelona Clinical Liver Cancer
CP: Child-Pugh
CSPH: Clinically significant portal pressure
HCC: Hepatocellular carcinoma
INR: International normalized ratio
MELD: Model of end-stage liver disease
OS: Overall survival
PHLF: Post-hepatectomy liver failure
