# Supplementary figures and images for: Ability of the ALBI grade to predict posthepatectomy liver failure and long-term survival after liver resection for different BCLC stages of HCC
Source: World J Surg Oncol. 2018 Oct 16;16:208. doi: 10.1186/s12957-018-1500-9 (PMC6192221; doi:10.1186/s12957-018-1500-9)

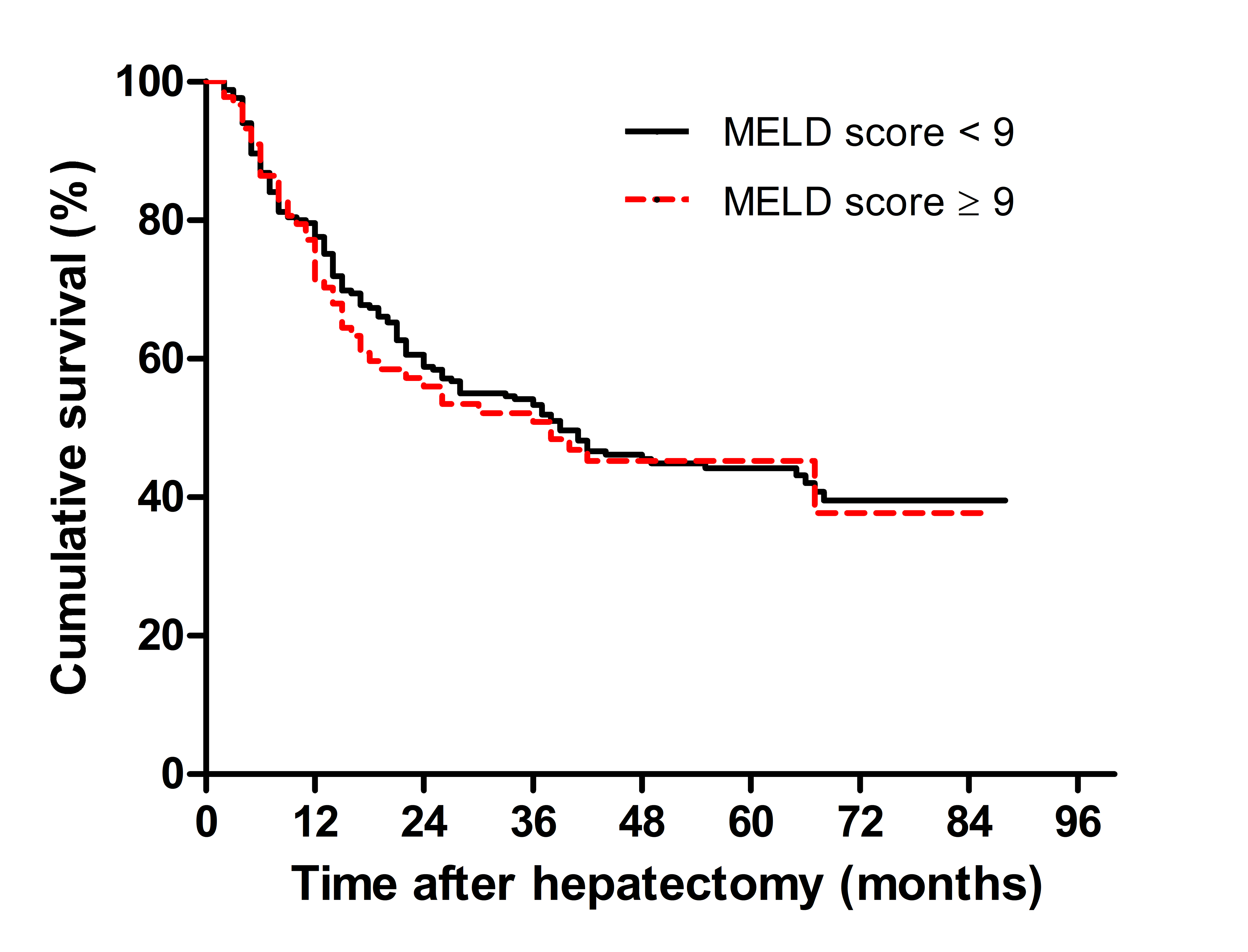

Supplement: Supplementary file 1 — Figure S1. Kaplan-Meier curves demonstrating overall survival according to MELD score < 9 and MELD score ≥ 9 in the total cohort. BCLC, Barcelona Clinic Liver Cancer; MELD, model for end-stage disease. (TIF 801 kb) [file 12957_2018_1500_MOESM1_ESM.tif]
